# Effects of *PPARG* and *PPARGC1A* gene polymorphisms on obesity markers

**DOI:** 10.3389/fpubh.2022.962852

**Published:** 2022-11-16

**Authors:** Carmen Muntean, Maria Oana Sasaran, Adriana Crisan, Claudia Banescu

**Affiliations:** ^1^Department of Paediatrics I, “George Emil Palade” University of Medicine, Pharmacy, Sciences, and Technology of Târgu Mureş, Târgu Mureş, Romania; ^2^Department of Paediatrics III, “George Emil Palade” University of Medicine, Pharmacy, Sciences, and Technology of Târgu Mureş, Târgu Mureş, Romania; ^3^Center for Advanced Medical and Pharmaceutical Research, “George Emil Palade” University of Medicine, Pharmacy, Science, and Technology of Târgu Mureş, Târgu Mureş, Romania

**Keywords:** *PPARG*, obesity, anthropometric parameters, metabolic parameters, child

## Abstract

Pediatric obesity presents a multifactorial etiology, which involves genetic traits as well, including single nucleotide polymorphisms. The aim of the study is to investigate the contribution of *PPARG* gene polymorphisms (namely Pro12Ala rs1801282, His447His rs3856806, and Pro115Gln rs1800571) and *PPARGC1A* rs8192678 SNP on the anthropometric and metabolic parameters in a population of Romanian children. We conducted a cross-sectional study of 295 Caucasian children, divided according to the body mass index (BMI) z-score into the study (obese and overweight) group of 130 children and the control (normoponderal) group of 165 children. Anthropometric parameters were greater in the obese and overweight population as opposed to controls, with significant differences (*p* < 0.01) found for the weight (2.77 ± 1.54 SD vs. −0.04 ± 1.15 SD), body mass index (BMI) (2.28 ± 0.97 SD vs. −0.18 ± 1.19 SD), mid-upper arm circumference (MUAC) (4.59 ± 2.28 SD vs. 0.28 ± 3.45 SD), tricipital skin-fold (TSF) (3.31 ± 3.09 SD vs. 0.62 ± 7.28 SD) and waist-to-height ratio (WHtR) (0.61 ± 1.51 SD vs. −0.35 ± 1.35 SD) z-scores. Moreover, triglyceride values were higher in the study group (118.70 ± 71.99 SD vs. 77.09 ± 37.39 SD). No significant difference in the allele and genotype distribution of investigates gene polymorphisms was observed between the studied groups (*p* > 0.05). *PPARG* (rs1801282, rs3856806, and rs1800571) were not associated with demographic, anthropometric, and laboratory parameters. However, *PPARGC1A* rs8192678 CC genotype was associated with TSF z-score (*p* = 0.03), whereas total and LDL cholesterol levels were significantly higher among TT homozygotes (*p* < 0.01). Our data suggest that *PPARG* (rs1801282, rs3856806, and rs1800571) and *PPARGC1A* (rs8192678) gene polymorphisms were not associated with childhood and adolescence overweight and obesity. The present study identified a significant increase in fasting glucose levels, triglyceride, albumin, and ALT levels in children with excess weight, as well as expected important upward variation of anthropometric parameters (BMI, MUAC, TSF z-scores).

## Introduction

In the last four decades, we faced a dramatic, ten-fold increase in obesity incidence. The World Health Organization (WHO) estimates that nearly 1 in 5 children and adolescents are overweight or obese (1). Nutritional status across Europe is miscellaneous, but a high prevalence of childhood obesity has been reported throughout the entire continent, especially in Southern Europe (2, 3). Overweight and obesity prevalence numbers in children from Romania are comparable to other European countries, with one recent study showing that every 1 in 4 individuals aged 6–19 years are overweight or obese (2, 4).

A multifactorial etiology stands behind excessive weight gain, ranging from high caloric intake to sedentary lifestyle, hormonal imbalance, gut microbiota imbalance, or hereditary factors (5). A lot of attention has been given in the past years to the implications of genetic backgrounds in the development of obesity, with studies evolving around various genes and different populations (6–8). Contradictory theories have emerged regarding the role of gene polymorphisms in the onset of childhood obesity, given that some single nucleotide polymorphisms (SNPs) constitute protecting factors in the development of multiple chronic diseases. Thus, associations between polymorphisms of genes such as leptin receptor (*LEPR*), fat mass obesity-associated receptors (*FTO*), melanocortin-4 receptors (*MC4R*), peroxisome proliferator-activated receptor-gamma (*PPARG*), and weight gain in school-aged children have been intensely studied (9–11).

Peroxisome proliferator-activated receptors, in particular, are abundantly expressed in adipose tissue, are one of the candidate genes in terms of metabolic regulation, by modulating adipocyte differentiation, lipid oxidation as well as glucose homeostasis, and insulin sensitivity (12, 13). There are multiple isoforms of PPAR gamma, namely γ1, γ2, and γ3. PPARγ1 and 3 are highly expressed in adipose tissue and at a lower level in other tissues as well (liver, colon, heart, etc) while PPARγ2 (which presents an additional 28 amino acids sequence on the N-terminal of PPARγ1) is expressed almost only in adipose tissue (14).

Mapped to chromosome 3p25.2 (Online Mendelian Inheritance in Man—OMIM number 601487), *PPARG* contains 9 exons and spans over more than 100 kb (12). Its transcription activity is reduced by a missense mutation, namely a proline12-to-alanine (Pro12Ala) substitution in the *PPARG* gene (rs1801282), close to the NH2-terminal region of the protein (15). Two of the most common variants in the *PPARG* gene are the ones of Pro12Ala (rs1801282 C > G), as well as of His447His (rs3856806 C>T) polymorphisms (16). Several studies conducted on the adult population showed a positive association between *PPARG* Pro12Ala polymorphism (rs1801282) and obesity in adults, ultimately proving the involvement of the variant G allele (17, 18). Furthermore, a systematic review also pointed out a relationship between the Pro12Ala polymorphism and an increase in body mass index (BMI) in healthy adults (19). His447His (rs3856806), also known as C161T, the other frequently encountered polymorphism of *PPARG*, seems to be involved in the maintenance of lipid metabolism equilibrium, with a possible indirect link to an increased risk of coronary heart disease (CHD), especially in patients with type 2 diabetes mellitus (T2DM) (20–22). Furthermore, Ristow et al. showed that the *PPARG* Pro115Gln (rs1800571) missense variant is associated with severe obesity, as it determines an increased activity of the gene, thus leading to enhanced adipocyte differentiation (23). Contradictorily, another study conducted by Bluher et al. concluded that rs1800571 SNP did not have an important role in the development of insulin resistance and obesity (24).

A transcriptional coactivator of PPARG, the peroxisome proliferator-activated receptor-G coactivator 1-alpha (PPARGC1A) also plays an essential role in regulating energy metabolism. PPARGC1A acts on the nuclear receptor PPARG, thus interacting with transcription factors (25). The GLy482Ser (rs8192678) SNP is a variant in the protein coding sequence of *PPARGC1A*, whose deleterious effect has been related to obesity, as well as its associated complications, which include CHD, T2DM, non-alcoholic fatty liver disease (NAFLD), and arterial hypertension (26).

To date, there are little data on the involvement of *PPARG* SNPs in the development of obesity in children, and reports on the implications of gene polymorphisms in the process and consequences of excessive weight gain are even scarcer in Romanian populations. Thus, the current study aims to investigate the contribution of three *PPARG* polymorphisms (namely Pro12Ala rs1801282, His447His rs3856806, and Pro115Gln rs1800571) and *PPARGC1A* rs8192678 SNP on the anthropometric and metabolic parameters in a population of Romanian children.

## Materials and methods

We conducted a prospective study on 295 Caucasian children aged between 3 months and 17 years, recruited within a timeframe of 36 months in a tertiary pediatric hospital from Tîrgu Mureş, Romania. Anthropometric parameters, biochemical traits, and SNPs of *PPARG2* and *PPARGC1A* were cross-sectionally assessed in each child, during the first visit. The division of the population sample among the two study groups (study group- obese, plus overweight and controls- weight within normal ranges) was dependent upon the values of the body mass index (BMI) z score, as recommended by the WHO. Thus, in children aged under 5 years, a z score between 1 and 2 standard deviations (SDs) defined risk of overweight, a z score of at least 2 SD was suggestive of overweight and a z score of at least 3 SD was interpreted as suggestive of obesity, whereas in children older than 5 years, a z score between 1 and 2 SDs was indicative of overweight and one exceeding 2 SDs translated into obesity (27, 28). Z score was applied instead of percentiles due to recent studies supporting its use over percentiles in classifying childhood overweight and obesity (29). Comparison of anthropometric parameters, biochemical traits, and *PPARG2* and *PPARGC1A* SNPs was therefore conducted between the two study groups.

Children included in the study were presented for regular check-ups or digestive complaints such as constipation, regurgitations, heartburn, abdominal pain, bloating. Patients with previously known chronic conditions or those who were diagnosed with food intolerances or chronic disorders of the digestive tract as a result of paraclinical investigations were excluded from the study.

### Anthropometric parameters

Anthropometric measurements were done by a single qualified person. Weight was measured using a properly calibrated scale, while height was quantified with the help of a calibrated length board/stadiometer, depending on the subject's age. BMI was calculated by dividing weight (kg) by squared height (in meters). Weight, height, waist-to-height ratio (WHtR), BMI, mid-upper arm circumference (MUAC), and tricipital skin-fold (TSF) z-scores were calculated in accordance with WHO growth charts. All anthropometric measurements (weight, height, waist circumference (WC), hip circumference (HC), MUAC, and TSF were obtained from shoeless children wearing lightweight clothing by applying a method that had been described by the authors in their previous research (9, 10). A novel parameter, which can assess excess weight and central obesity status, namely waist-to-height ratio (WHtR) was also calculated and interpreted according to recent recommendations: normal values < 0.45, overweight for values ≥0.45, and obesity for values exceeding 0.5 (30).

### Biochemical variables

Serum biochemical and metabolic traits from venous blood sample (glucose, total cholesterol (TC), high-density lipoprotein cholesterol (HDL-c), low-density lipoprotein cholesterol (LDL-c), triglycerides (TG), liver enzymes, total protein levels, fasting glucose levels, serum albumin) were assessed using standard laboratory methods (with the help of a Cobas Integra 6000 plus automated analyzer, Roche Diagnostics GmbH, Mannheim, Germany).

### DNA extraction and genotyping

For DNA extraction 2 mm of venous blood were collected on tubes with Ethylenediamine tetraacetic acid (EDTA) as anticoagulated from each child included in the study. PureLink Genomic DNA Mini Kit (Invitrogen, Thermo Fischer Scientific, USA) was used to obtain genomic DNA. Genotyping of *PPARG* rs1801282, rs3856806, and rs180057 and *PPARGC1A* rs8192678 polymorphism were determined through PCR (polymerase chain reaction) followed by digestion with the restriction enzyme (RFLP restriction fragment length polymorphism method). The PCR conditions, primers, and restriction enzymes used were according to previously described protocols (31–34).

### Statistical analysis

Statistical analysis was done using GraphPad Prism T software, version 9.0. Descriptive statistics were applied to quantitative variables and expressed as mean ± standard deviation (SD). The frequency of categorical variables was expressed in percentages (%). The number and frequency distributions of genetic polymorphisms were assessed using the Poisson random field (PRF) model (35). Shapiro-Wilk test was used to assess distribution characteristics of analyzed data. Consequently, mean comparison was conducted using the Mann-Whitney test for non-Gaussian distributed variables and Student's unpaired *t*-test for variables complying with a Gaussian distribution pattern. Comparison of multiple data sets was performed with the help of analysis of variance (ANOVA), namely after applying the non-parametric Kruskal-Wallis test. Categorical variables such as sex, genotype, allele frequency, as well as the Hardy-Weinberg equilibrium, were analyzed using the Chi-square test. Mul-tiple linear regressions were applied to assess the associ-ation between genotype frequency and children's BMI, on which the division of the study groups was conducted, while also taking different confounders into consideration. Adjustment for confounding factors consisted of two models, namely model A, in which maternal age, parity, weight (devised according to BMI), educational level and ethnicity was taken into account, and model B, in which paternal age, weight (devised according to BMI) and ethnicity were considered. The level of significance was set at *p* < 0.05, corresponding to a confidence interval of 95%.

## Results

The study cohort consisted of 295 Caucasian children, who had a mean age of 10.24 ± 4.38 years and were divided in accordance with the BMI z score into two groups, a study (obese and overweight) group of 130 children (85 obese and 45 overweight children) and a control (normoponderal) group of 165 children. The comparison of demographic, anthropometric, and metabolic data of all subjects are presented in Table 1. There was no significant difference between the two groups in terms of mean age, but the male sex was significantly more prevalent among the study group (*p* < 0.01, OR = 2.10). Anthropometric parameters were expectably greater in the study population as opposed to controls, with significant differences (*p* < 0.01) found for weight (2.77 ± 1.54 SD vs. −0.04 ± 1.15 SD), BMI (2.28 ± 0.97 SD vs. −0.18 ± 1.19 SD), MUAC (4.59 ± 2.28 SD vs. 0.28 ± 3.45 SD), TSF (3.31 ± 3.09 SD vs. 0.62 ± 7.28 SD) and I/L (0.61 ± 1.51 SD vs. −0.35 ± 1.35 SD) z scores. The lipid profile comparison showed insignificant variation between the two groups of total HDL and LDL cholesterol levels, but triglyceride values were higher in the study group (118.70 ± 71.99 SD vs. 77.09 ± 37.39 SD). An ascending trend was also found for basal glucose (91.66 ± 19.33 SD vs. 83.13 ± 10.75 SD), ALT (31.18 ± 36.49 SD vs. 22.30 ± 15.86 SD), and total protein levels (4.64 ± 0.50 SD vs. 4.35 ± 0.55 SD) in obese and overweight children.

**Table 1:** Comparison of demographic, anthropometric, metabolic, and paraclinical data of the study population.

**Parameter**	Control group*(*n* = 165)	Study group*(*n* = 130)	***p*-value**
Mean age	9.83 ± 4.1	10.5 ± 4.58	0.15
Female sex (%)	35.59	20	< 0.01
Male sex (%)	20.34	24.07	
Weight—z score	−0.04 ± 1.15	2.77 ± 1.54	< 0.01
BMI—z score	−0.18 ± 1.19	2.28 ± 0.97	< 0.01
MUAC—z score	0.28 ± 3.45	4.59 ± 2.28	< 0.01
TSF—z score	0.62 ± 7.28	3.31 ± 3.09	< 0.01
WHtR—z score	−0.35 ± 1.35	0.61 ± 1.51	< 0.01
Total cholesterol level (mg/dl)	160.2 ± 28.74	163.4 ± 38.92	0.80
HDL—c level (mg/dl)	52.92 ± 13.63	51.27 ± 14.42	0.16
LDL—c level (mg/dl)	95.21 ± 30.97	90.71 ± 31.25	0.08
Triglycerides (mg/dl)	77.09 ± 37.39	118.70 ± 71.99	< 0.01
AST (U/l)	28.56 ± 11.93	28.68 ± 18.55	0.42
ALT (U/l)	22.30 ± 15.86	31.18 ± 36.49	< 0.01
Glucose level (mg/dl)	83.13 ± 10.75	91.66 ± 19.33	< 0.01
Total protein level (g/dl)	7.41 ± 0.69	7.57 ± 0.50	0.10
Albumin level (g/dl)	4.35 ± 0.55	4.64 ± 0.50	< 0.01

^*^Data is listed as mean ± SD for absolute variables and percentage (%) for categorical variables (female/male sex).

AST, aspartate aminotransferase; ALT, alanine aminotransferase; BMI, body mass index; HDL-c, high-density lipoprotein cholesterol; LDL-c, low-density lipoprotein cholesterol; MUAC, medium upper arm circumference; OR, odds ratio; SD, standard deviation; TSF, tricipital skin fold; WHtR, waist-to-height ratio.

The genotype frequencies of the investigated *PPARG* (rs1801282, rs3856806, rs1800571) and *PPARGC1A* rs8192678 polymorphisms were in agreement with the Hardy–Weinberg equilibrium (*p* < 0.05). The comparative assessment between the two study groups of genotype and allele frequency for the four studied polymorphisms can be visualized in Table 2. Comparison of *PPARG* genotype and allele frequency for the three studied polymorphisms, namely rs1801282, rs3856806, rs1800571, revealed no significant dissimilarities. The rs1800571 (Pro115Gln) SNP was distinguished among others through an identification of the wild type homozygous genotype or the wild type allele among nearly every subject included in the study (Table 2). Assessment of the rs8192678 SNP of *PPARGC1A*, revealed the dominant frequency of the C allele within both the study and the control group but without a significant difference.

**Table 2:** Comparison of the genotype and allele frequencies of investigated SNPs within the entire study cohort, between the study (overweight/obese) group and control group.

**Gene**	**Investigated SNP**		**Genotype frequency (%)**			***p-*value**	**Allele frequency (%)**		***p*-value**
*PPARG*	rs1801282 (Pro12Ala)		CC	CG	GG		C	G*	
		Study group (*n* = 130)	78.46	18.46	3.07	0.99	87.69	12.3	0.99
		Control group (*n* = 165)	78.18	19.39	2.42		87.87	12.12	
		Entire study population (*n* = 295)	78.3	18.98	2.71		87.79	12.2	
	rs1800571 (Pro115Gln)		CC	CA	AA		C	A*	
		Study group (*n* = 130)	98.46	1.53	0	0.27	99.23	0.76	0.27
		Control group (*n* = 165)	100	0	0		100	0	
		Entire study population (*n* = 295)	99.32	0.67	0		99.66	0.33	
	rs3856806 (His447His)		CC	CT	TT		C	T*	
		Study group ( *n* = 130)	74.61	22.3	3.07	0.96	85.76	14.23	0.81
		Control group (*n* = 165)	78.18	18.78	3.03		87.57	12.42	
		Entire study population (*n* = 295)	76.61	20.33	3.05		86.77	13.22	
*PPARGC1A*	rs8192678 (Gly482Ser)		CC	CT	TT		C	T*	0.21
		Study group (*n* = 130)	44.61	40.76	14.61	0.79	65	35	
		Control group (*n* = 165)	39.39	40.6	20		59.69	40.3	
		Entire study population (*n* = 295)	41.69	40.67	17.62		62.03	37.96	

^*^Variant allele, PPARG, peroxisome proliferator-activated receptor gamma.

Ethnicity, maternal and paternal characteristics, including age, weight (devised according to BMI), parity and educational level were compared between the two study groups (Table 3). There were no significant differences between the two groups in terms of parental age. However, parents of children belonging to the study group presented significantly higher prevalence of overweight and obesity. High maternal educational level and low parity represented protective factors against abnormally high child BMI (*p* < 0.01), whereas Roma ethnicity were more prone to overweight and obesity (*p* < 0.01, OR = 3.04). Race, maternal and parental characteristics were afterwards considered as confounding factors for assessment of correlation between genotype of the studied SNPs and patient's BMI. Multiple linear regression was applied using two different models, as further detailed in Table 4, but no significant results were obtained.

**Table 3:** Comparison of ethnicity, parental weight, age, maternal, parity and educational level between the two study groups.

		Control group*(*n* = 165)	Study group*(*n* = 130)	***p*-value**
Ethnicity	Caucasian (%)	48.81	30.5	< 0.01
	Roma (%)	7.11	13.55	
Maternal age	36.22 ± 7.22	36.24 ± 4.93	0.69
Maternal weight	Normal (%)	42.37	17.28	< 0.01
	Overweight (%)	13.55	17.62	
	Obese (%)	0	9.15	
Maternal educational level	Low (%)	7.79	11.52	< 0.01
	Middle (%)	31.52	24.4	
	High (%)	16.65	8.13	
Maternal parity	1 (%)	27.45	10.5	< 0.01
	2 (%)	24.06	18.64	
	≥3 (%)	4.40	14.91	
Paternal age	38.59 ± 8.19	37.69 ± 5.71	0.35
Paternal weight	Normal (%)	41.35	22.71	< 0.01
	Overweight (%)	11.18	19.66	
	Obese (%)	0	1.69	

^*^Data is listed as mean ± SD for absolute variables and percentage (%) for categorical variables.

Normal weight: BMI < 25 kg/m^2^, overweight when 25 ≤ BMI < 30 kg/m^2^ and obese: BMI > 30 kg/m^2^; educational level: low, lack of high-school diploma, middle, obtainment of high school diploma, high, obtainment of college/university degree.

**Table 4:** Multiple linear regression for association between genotype frequency and patients' BMI, after adjustment for parental confounders using two models.

**Model**	**Gene (investigated SNP)**	**Genotype**	**Coefficient**	**Standard error**	**95 % CI**	***t***	***p*-value**	**VIF**
Model A		
	PPARG rs1801282 (Pro12Ala)	CC-ref.
		CG	−0.09	0.26	−0.61–0.42	0.35	0.71	1.16
		GG	0.18	0.67	−1.13–1.50	0.27	0.78	1.06
	PPARG rs1800571 (Pro115Gln)	CC-ref.	
		CA	−0.24	1.15	−2.52–2.02	0.21	0.83	1.07
	PPARG rs3856806 (His447His)	CC-ref.	
		CT	−0.04	0.26	−0.55–0.46	0.17	0.85	1.18
		TT	0.94	0.59	−0.22–2.11	1.59	0.11	1.10
	PPARGC1A rs8192678 (Gly482Ser)	CC-ref.	
		CT	−0.08	0.22	−0.52–0.35	0.38	0.69	1.28
		TT	0.11	0.28	−0.44–0.66	0.40	0.68	1.26
Model B								
	PPARG rs1801282 (Pro12Ala)	CC-ref.	
		CG	−0.30	0.32	−0.94–0.33	0.93	0.34	1.16
		GG	−0.23	0.79	−1.80–1.32	0.30	0.76	1.02
	PPARG rs1800571 (Pro115Gln)	CC-ref.	
		CA	2.00	1.36	−0.68–4.68	1.46	0.14	1.02
	PPARG rs3856806 (His447His)	CC-ref.	
		CT	0.11	0.31	−0.51–0.74	0.36	0.71	1.15
		TT	0.94	0.71	−0.45–2.35	1.32	0.18	1.09
	PPARGC1A rs8192678 (Gly482Ser)	CC-ref.	
		CT	−0.18	0.27	−0.71–0.34	0.68	0.49	1.26
		TT	0.02	0.34	−0.65–0.70	0.06	0.94	1.20

CI, confidence interval; Model A, adjustment in accordance to maternal age, parity, weight (devised according to BMI), educational level, and ethnicity; Model B, adjustment in accordance to paternal age, weight (devised according to BMI), and ethnicity; ref., reference (the homozygous genotype of the wild allele was selected as reference); VIF, variance inflation factor.

Comparison of demographic and laboratory parameters presented in Table 1 was also conducted by dividing the entire study population, as well as the obese/overweight group by genotype distribution. This analysis did not include rs1800571, due to the very limited number of subjects with variant heterozygous genotypes and the absence of subjects with variant homozygous genotypes. Analyzed parameters presented similar mean values, regardless of genotype in the case of *PPARG* rs1801282, rs3856806 SNPs. Genotype-based separation of demographic and paraclinical data showed no statistical significance for any of the studied parameters (Table 5). For *PPARGC1A*, the same analysis was performed by dividing the study subjects between CC homozygous genotype and both variant homozygous and heterozygous genotypes. Total and LDL cholesterol levels were significantly higher among TT homozygotes (*p* < 0.01).

**Table 5:** Comparison of demographic, anthropometric, metabolic, and paraclinical data of the overweight/obese group divided in accordance with genotype distribution of *PPARGC1A* rs8192678 SNP.

**Parameter**	**CC (*n* = 58)**	CT *(*n* = 53)	TT*(*n* = 19)	***p*-value**
Age(years)	9.67 ± 4.32	9.83 ± 3.69	10.35 ± 4.67	0.83
Female sex (%)	19.23	17.69	8.46	0.62
Male sex (%)	25.38	23.07	6.15	
Weight—SD	2.78 ± 1.49	2.67 ± 1.28	3.00 ± 2.30	0.95
BMI—z score (kg/m^2^)	2.09 ± 0.88	2.49 ± 1.00	2.28 ± 1.09	0.29
MUAC—z score	4.41 ± 2.16	4.76 ± 2.03	4.74 ± 3.22	0.90
TSF—z score	3.17 ± 3.16	3.58 ± 3.25	3.02 ± 2.43	0.43
Total cholesterol level (mg/dl)	168.30 ± 44.51	156.40 ± 29.77	168.20 ± 42.15	0.31
HDL cholesterol level (mg/dl)	49.98 ± 14.54	51.60 ± 13.88	54.25 ± 15.77	0.56
LDL cholesterol level (mg/dl)	90.71 ± 36.10	87.67 ± 23.68	101.60 ± 36.31	0.42
Triglycerides (mg/dl)	121.30 ± 75.25	117.80 ± 71.93	113.70 ± 64.92	0.98
Fasting glucose level (mg/dl)	93.50 ± 23.26	90.44 ± 15.83	89.36 ± 14.61	0.87
AST (U/l)	29.13 ± 14.20	29.61 ± 24.26	24.58 ± 8.10	0.74
ALT (U/l)	31.75 ± 25.50	33.23 ± 49.23	23.95 ± 15.78	0.47
Total protein level (g/dl)	7.57 ± 0.50	7.58 ± 0.50	7.55 ± 0.51	0.90
Albumin level (g/dl)	4.56 ± 0.52	4.72 ± 0.43	4.64 ± 0.58	0.33

^*^Data is listed as mean ± SD for absolute variables and percentage (%) for categorical variables (female/male sex).

AST, aspartate aminotransferase; ALT, alanine aminotransferase; BMI, body mass index; HDL-c, high-density lipoprotein cholesterol; LDL-c, low-density lipoprotein cholesterol; MUAC, medium upper arm circumference; OR, odds ratio; SD, standard deviation; TSF, tricipital skin fold.

The other parameters showed no significant allele-dependent variation, although an ascending trend can be seen for mean anthropometric constants, as well as for liver enzyme, total protein, albumin, triglyceride values, and fasting glucose levels. Furthermore, homozygotes for C allele presented higher mean values of the TSF (*p* = 0.03). The same type of analysis was carried out in the obese/overweight population group without obtaining statistically significant results.

**Figure 1:**
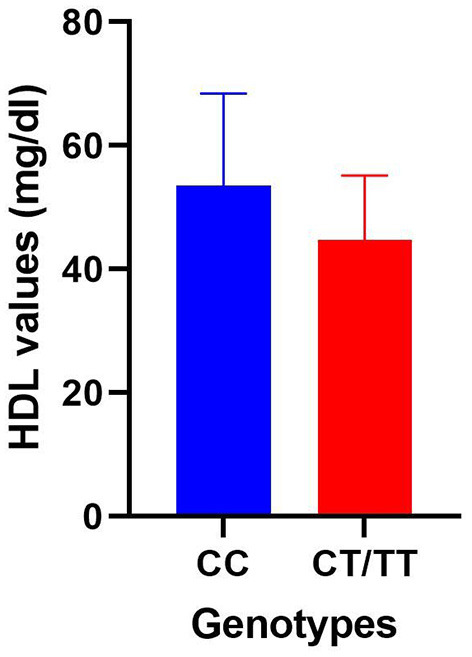
Comparison of HDL mean values within the obese/overweight group divided between homozygous CC genotype and a group consisting of variant heterozygous and homozygous genotypes for the *PPARGC1A* rs3856806 polymorphism.

Allele-based comparisons were also carried out both for *PPARG* rs1801282 (CC vs. CG + GG) and rs3856806 (CC vs. CT + TT) SNPs within the entire study population and within the obese/overweight group but analyzed parameter variation was insignificant with only one exception. An important increase in mean HDL levels was associated with homozygous CC genotype in the case of PPARG rs3856806 SNP (53.47 ± 14.96 SD vs. 44.77 ± 10.42 SD, *p* < 0.01), as illustrated in Figure 1.

A comparison of obesity/anthropometry parameters between subjects with concomitant presence of variant alleles and those with wild-type alleles was conducted for those three genotypes included in the study (PPARG rs1801282, PPARG rs3856806, and PPARGC1A rs8192678) in which an important prevalence of the variant allele was encountered. No significant discrepancies were found. Therefore, separate, similar, individual comparisons of anthropometric and obesity parameters were carried out for two polymorphisms of those three aforementioned. An important decrease in HDL levels was linked to the concomitant presence of variant alleles of PPARG rs3856806 and PPARGC1A rs8192678 (44.79 ± 10.23 SD vs. 51.97 ± 15.34 SD, *p* = 0.04). Variant allele carriers of both PPARG rs1801282 and PPARGC1A rs8192678 exhibited higher mean TSF values (3.96 ± 2.29 SD vs. 3.23 ± 3.35 SD, *p* = 0.03). Other similar comparisons for other parameters or the other two combinations of genotypes (PPARG rs1801282 and PPARG rs3856806) yielded insignificant results.

## Discussion

Childhood and adolescence obesity can determine metabolic complications that persist through adulthood and translate into cardiovascular risk factors (36). Dyslipidemia, a predictor of atherosclerosis, is greatly influenced by physical activity level, dietary or genetic factors (37). It has been associated with anthropometric parameters, with many studies confirming a relationship between measured constants which are suggestive of an excessively represented adipose tissue, such as BMI, MUA, TSF, WC or HC (38). Still, the term “metabolically healthy obesity” (MHO) has recently emerged, and defines obese subjects whose triglycerides, high-density lipoprotein cholesterol, blood pressure, and fasting glucose levels are within normal ranges. Its prevalence has been reported at 31% in one pediatric study, but varies in accordance to definitions used and types of parameters evaluated (39, 40). In our study, we found no significant differences in terms of total cholesterol, HDL- or LDL-cholesterol values, but triglycerides presented an ascending trend in children with obesity as opposed to the normoponderal ones. The same pattern was observed for fasting glucose levels, in resonance with other studies, which also sustain the proven effect of weight loss on basal glucose values (41, 42). Moreover, a significant increase in ALT values was found in children with obesity, which might constitute an important, yet not single marker of childhood NAFLD (43).

Research regarding the involvement of SNPs in pediatric metabolic imbalances is still ongoing. SNPs seem to have an impact on glucose hemostasis, lipid metabolism, anthropometric parameters, leptin and adiponectin levels in subjects with obesity, starting from an early age (44–47). Several recent studies have highlighted the influence of SNPs in childhood obesity in Romania, with particular focus on *FTO* rs9939609, *LEPR* 223, and 1019 gene polymorphisms, interleukin 6 (*IL-6)* 572 C/G, 190 C/T and angiotensin-converting enzyme gene insertion/deletion (*ACE* I/D) gene polymorphisms (9, 10, 48, 49). However, the impact of *PPARG* SNPs on the obesity risk in children has been scarcely studied, literature data reporting mostly associations with adulthood obesity and its related complications (26, 50). *PPARG* gene expression has been negatively correlated with IL-6 and TNF-α, which are known to play an important role in regulating inflammatory status related to pediatric obesity (51). Furthermore, *PPARG* rs1801282 polymorphism has been controversially linked to a decrease in BMI, according to a study conducted in Portugal (11). Other research data supported an association between *PPARG* Pro12Ala (rs1801282) polymorphism and insulin resistance in Mexican children with dyslipidemia (52). Hence, the role of *PPARG* polymorphism in pediatric weight gain and its related complications still remains unclear.

Through this study we tried to investigate a possible association between three polymorphisms of the *PPARG* gene, as well as one polymorphism of the *PPARGC1A* gene and pediatric obesity and overweight.

Pediatric studies have focused on *PPARG* mostly. A very recent study in teenagers from the North of Mexico revealed that carriers of the *PPARG* G allele (Pro12Ala or rs1801282) present an increased risk for high WHtR or LDL-c (53). These results expectingly contradict our conclusions, in light of a scarcity of homozygous GG genotype within our study. The very low prevalence of homozygous genotype Ala/Ala (GG) frequency in obese and overweight children is in resonance with the one reported by Aiman et al. in their study that did not identify a single subject with a GG genotype for the *PPARG* Pro12Ala polymorphism. Moreover, the authors found a significant association between Pro/Pro (CC), heterozygous Pro/Ala (CG) carriers of Pro12Ala *PPARG* gene variant and a higher mean BMI, WC, and HC in overweight and obese Malay children population (54). On the other hand, in our research, the C allele (Pro12Ala or rs1801282 polymorphism) (although more prevalent as well within our study population) did not exhibit an influence upon anthropometric or biochemical parameters.

In addition, we investigated the presence of at least 2 variant genotype for *PPARG* (rs1801282, rs3856806, and rs1800571) polymorphisms in investigated children, in an attempt to assess their contribution in the determinism of excess weight but no relevant results were obtained, even after adjusting for potential parental confounders.

Polymorphism variation is dependent upon race, as proven by a nationwide study involving pre-school children from the United States of America which reported a higher prevalence of obesity among Hispanic populations as in Caucasians (55). This might explain miscellaneous results obtained by various authors which prove a lack of association between genetic SNPs of multiple genes, including *PPARG* and obesity. Queiroz et al. reported no association for *PPARG* rs1801282 and several anthropometric and metabolically-related parameters (BMI, WC, total cholesterol, LDL/HDL cholesterol, insulin, and HOMA-IR, body fat percentage, birth weight, systolic blood pressure), within a research article conducted on Brazilian children and adolescents (56). Two other reports described an insignificant impact of *PPARG* rs3856806 polymorphism on BMI value variations among children and adolescents (57, 58). In our study we failed to identify an association between three polymorphisms of *PPARG* and children with overweight and obesity. Our results are similar to those reported by Clement et al., whose study highlighted the lack of correlation between *PPARG* rs1800571 (Pro115Gln), rs1801282 (Pro12Ala) variants and obesity in French Caucasians. Furthermore, the study underlined the absence of the Pro115Gln mutation in the entire study population (59). Considering the low number of patients with rs1800571 variant (only two heterozygotes) within our study groups as well, the significance of this mutation within Caucasian populations is raised into question. Nevertheless, no association was found in another Portuguese study between *PPARG* polymorphisms and obesity in premenopausal women, which concludes this SNP may differ between populations and probably should not be considered as a strong genetic marker to evaluate risk for obesity as *FTO* rs9939609 has been reported (60).

Relevant associations between metabolic parameters, lipid profile, and *PPARG* polymorphisms have though been found, even among studies that could not conclude that the analyzed gene variants represent an obesity/overweight risk. Particularly, Vidovic et al. found lower mean values of HDL-c in the CT and TT genotype of *PPARG* rs3856806, despite of a lack of association between the aforementioned polymorphism and overweight/obesity among Serbian adolescents (57). These findings closely resemble results obtained in our study, in which CC genotype (*PPARG* rs3856806) was significantly associated with higher mean HDL-c values and carriers of variant alleles of both PPARG rs3856806 and PPARGC1A rs8192678 presented significantly lower serum HDL-c levels. Still, the authors also report higher fasting blood glucose levels within the same CT and TT genotype group (57), which we could not trace in our study. Contradictorily, Fan et al. reported a correlation between minor allele variants of rs3856806 and elevated LDL-c levels in Chinese adults (61). Once again, these miscellaneous results prove the race variation of genetic polymorphisms.

A previous study on unrelated German subjects demonstrated that the *PPARG* Pro115Gln (rs1800571) missense variant is possibly pathogenic for severe obesity (23). On the other hand, Hamer et al. claimed that the Pro115Gln polymorphism does not epidemiologically influence the prevalence of morbid obesity (62). Similarly, the same polymorphism did not seem to be related to obesity in Malaysian teenagers (63). Furthermore, the rs1800571 SNP did not contribute considerably to insulin resistance and obesity occurrence, according to Blüher et al. (24). In our research, variant allele of the *PPARG* rs1800571 was found in a very small number of subjects, in accordance with literature data performed on Caucasian populations. The presence of the *PPARG* rs1800571 variant allele in only 2 subjects supports results obtained by Ek et al. and Bidzinska et al. in studies conducted on Caucasian, European populations, which failed to identify the Pro115Gln mutation in any of the enrolled subjects (64, 65). Furthermore, this mutation has been classified as part of the rare sequence variants by a study conducted in South Africa (66). Thus, all these data sustain the theory that this variant is highly unlikely to be involved in the pathogenesis of obesity.

In our geographical area, research on *PPARG* and obesity has been limited so far to two studies. One of them is a cross-sectional study which proved that the wild-type C allele of the *PPARG* rs1801282 gene polymorphism is associated with an increased risk of childhood obesity in the offspring of obese mothers (67). Moreover, a second study conducted on Romanian and Moldavian children underlined the lack of clinical impact of *PPARG* gene polymorphism on insulin resistance among an overweight population sample (4). Hence, research on *PPARG* polymorphisms in Romania is scarce and few information is available regarding variant allele carriers in healthy populations.

We identified no significant association between the *PPARGC1A* Gly482Ser polymorphism and a study group consisting of overweight and obese children, as suggested by the significantly higher prevalence of the C allele. Still, for the same polymorphism, significantly higher mean TSF values were correlated with the CC homozygotes, as opposed to the CT and TT genotypes. On the other hand, higher mean TSF values were also encountered in those patients which were variant carriers of both G allele and T allele of PPARG rs1801282 and PPARGC1A rs8192678, respectively. Although anthropometric and biochemical parameters were not associated with any of the rs8192678 polymorphism genotypes, total and LDL cholesterol levels were higher among homozygous variant (T) allele carriers, similarly with the results obtained in another Romanian study, which enrolled an adult population affected by metabolic syndrome (34). Still, this polymorphism has been linked to variations in HDL-c concentrations among obese subjects, as well as with insulin resistance within an obese population (68, 69). Other studies have reported a lack of association between *PPARGC1A* Gly482Ser polymorphism and obesity or a decreased metabolic risk for those with 482Gly/Gly genotype (70, 71). It is important though to mention that these studies have involved only adults, pediatric research articles reporting limited data regarding *PPARGC1A* polymorphisms, mainly a relationship between this gene's A risk allele and NAFLD in obese children (72).

A recent study found that PPRAG rs1801282 modified the association between *n*-3 long-chain PUFA (LCPUFA) and serum insulin with an inverse association in minor allele (Ala12 = G) carriers only and no interaction between fatty acid desaturase *FADS* or *n*-3 LCPUFA and plasma glucose (73). This is in agreement with the results of previous research, where fish oil reduced plasma glucose only in carriers of the rs1801282 G allele in the PPRG2 gene (74). Damsgaard et al. (73) found an inverse association between seric *n*-3 LCPUFA and triglycerides only in the *PPARG2* rs1801282 G allele carriers, which concurs with the results of a previous study in infants (74). Blood *n*-3 LCPUFA was positively associated with LDL-c in children who were C allele homozygous of the rs1801282 SNP in the *PPARG2* gene (73).

Another study found that T allele carriers of *PPARGC1A* rs8192678 had a higher decrease in TC and LDL-c levels following a low-fat diet compared to CC homozygotes (75) in overweight and obese Spanish subjects. A similar trend was noticed in the A allele carriers vs. CC homozygotes in those with a moderately high-protein diet group. A significant decrease in the rate/percentage of hypercholesterolemia was observed just in CC homozygotes who underwent the moderately high-protein diet and not in the low-fat diet group (75). Furthermore, this study conducted by Ramos-Lopez et al. indicated that an energy-restricted moderately high-protein diet yielded better results in decreasing serum cholesterol among *PPARGC1A* CC homozygotes than a low-fat diet (75).

PPARG polymorphisms have also been studied in relation to aerobic training response. In a previous review that studied the effectiveness of PPARG and their coactivators' polymorphism genes' influence on human training response, the cases with TT *PPARGC1A* rs8192678 genotype were non-responders for aerobic training while those with CC *PPARGC1A* rs8192678 genotype were the best responders to aerobic training (76). Moreover, the presence of C allele for *PPARG* rs1801282 carriers has been associated with a better response to exercise as well as to nutritional intervention (76). The same meta-analysis found that subjects with *PPARG* rs1801282 CC genotype are the negative responders to aerobic training (decreased fasting insulin response and glucose tolerance after aerobic training). In their study, Zarebska et al. found that the *PPARG* genotype can modulate training response and induced/produced body mass and composition parameters changes (77). They found that young Polish women with CC *PPARG* rs1801282 genotype had a better response for body fat mass variables compared to G allele carriers after a 12-week training program (77). However, data on this previously debated matter is scarce in the pediatric population and has been so far limited to adolescents, in whom a physical exercise intervention might imply a better compliance as opposed to younger ages (78).

The main strength of our study was the assessment of three different polymorphisms of *PPARG*, which were not simultaneously analyzed before in Romanian children, as well as the evaluation of *PPARGC1A* in the same subjects, only briefly discussed in pediatric literature. The relatively small sample size for a study of this type, as well as the unicentric enrollment character of the study constitute limitations of our research which cannot be neglected and might have influenced our results. A low statistical power has been noted through a post ad-hoc analysis particularly within the combined anthropometric parameter comparisons of three of the studied polymorphisms. Moreover, we did not investigate a possible influence of PPARG polymorphisms on nutritional intervention and exercise benefits in the obese/overweight study group, as follow-up of subjects included in the study would have implied a longitudinal design, particularly hard to achieve at young ages. Still, a clearer image of *PPARG* and *PPARGC1A* polymorphisms involved in childhood obesity most likely requires further studies performed in various geographical regions.

## Conclusion

Genetic traits of obesity represent an attractive research topic in children, given the challenges faced by future metabolic-related complications and the limited literature data on the subject. Our findings showed no association of variant genotypes of *PPARG* (rs1801282, rs3856806, and rs1800571) and *PPARGC1A* (rs8192678) gene polymorphisms with childhood and adolescence overweight and obesity. Our study identified a significant increase in fasting glucose levels, triglyceride, albumin, ALT levels in children with excess weight, as well as expected important upward variation of anthropometric parameters (BMI, MUAC, and TSF z scores).

The limited number of patients, enrolled from a single referral center is a limitation of our study which once again highlights the need of expanding future research on *PPARG, PPARGC1A* SNPs and other polymorphisms on pediatric populations from various parts of the world. Therefore, the importance of race and geographical region cannot be overseen in studies evolving around clinical impact of polymorphism in weight gain.

## Data availability statement

The original contributions presented in the study are included in the article/supplementary materials, further inquiries can be directed to the corresponding authors.

## Ethics statement

The studies involving human participants were reviewed and approved by Institutional Ethics Committee of the George Emil Palade University of Medicine, Pharmacy, Sciences, and Technology of Târgu Mureş (Institutional Review Board no. 13/18 July 2011). Written informed consent to participate in this study was provided by the participants' legal guardian/next of kin.

## Author contributions

CM and CB contributed to the design of the study. MOS performed the statistical analysis. AC and CB carried out genetic analyses. CM, MOS, AC, and CB were involved to interpret the data. CM, MOS, and CB wrote and reviewed the manuscript. All authors read and approved the final version of the manuscript.

## Funding

This work was partly supported by a project financed by the Romanian Ministry of Education and Research, CNCS-UEFISCDI, Project No PN-III-P4-ID-PCE-2020-1928, within the PNCDI III, Contract No. PCE 72/2021.

## Conflict of interest

The authors declare that the research was conducted in the absence of any commercial or financial relationships that could be construed as a potential conflict of interest.

## Publisher's note

All claims expressed in this article are solely those of the authors and do not necessarily represent those of their affiliated organizations, or those of the publisher, the editors and the reviewers. Any product that may be evaluated in this article, or claim that may be made by its manufacturer, is not guaranteed or endorsed by the publisher.
